# Pancreatic stellate cells contribute pancreatic cancer pain via activation of sHH signaling pathway

**DOI:** 10.18632/oncotarget.7776

**Published:** 2016-02-27

**Authors:** Liang Han, Jiguang Ma, Wanxing Duan, Lun Zhang, Shuo Yu, Qinhong Xu, Jianjun Lei, Xuqi Li, Zheng Wang, Zheng Wu, Jason H. Huang, Erxi Wu, Qingyong Ma, Zhenhua Ma

**Affiliations:** ^1^ Department of Hepatobiliary Surgery, First Affiliated Hospital of Xi'an Jiaotong University, Xi'an 710061, China; ^2^ Department of Anesthesiology, First Affiliated Hospital of Xi'an Jiaotong University, Xi'an 710061, China; ^3^ Department of General Surgery, First Affiliated Hospital of Xi'an Jiaotong University, Xi'an 710061, China; ^4^ Department of Neurosurgery, Baylor Scott and White Health Care, Temple, TX, 76508, USA; ^5^ Department of Surgery, Texas A & M College of Medicine, Temple, TX, 76504, USA; ^6^ Department of Pharmaceutical Sciences, Texas A & M Health Science Center, College Station, TX, 77843, USA

**Keywords:** pancreatic cancer, pain, pancreatic stellate cells, sHH

## Abstract

Abdominal pain is a critical clinical symptom in pancreatic cancer (PC) that affects the quality of life for PC patients. However, the pathogenesis of PC pain is largely unknown. In this study, we show that PC pain is initiated by the sonic hedgehog (sHH) signaling pathway in pancreatic stellate cells (PSCs), which is activated by sHH secreted from PC cells, and then, neurotrophic factors derived from PSCs mediate the pain. The different culture systems were established *in vitro*, and the expression of sHH pathway molecules, neurotrophic factors, TRPV1, and pain factors were examined. Capsaicin-evoked TRPV1 currents in dorsal root ganglion (DRG) neurons were examined by the patch-clamp technique. Pain-related behavior was observed in an orthotopic tumor model. sHH and PSCs increased the expression and secretion of TRPV1, SP, and CGRP by inducing NGF and BDNF in a co-culture system, also increasing TRPV1 current. But, suppressing sHH pathway or NGF reduced the expression of TRPV1, SP, and CGRP. *In vivo*, PSCs and PC cells that expressed high levels of sHH could enhance pain behavior. Furthermore, the blockade of NGF or TRPV1 significantly attenuated the pain response to mechanical stimulation compared with the control. Our results demonstrate that sHH signaling pathway is involved in PC pain, and PSCs play an essential role in the process greatly by inducing NGF.

## INTRODUCTION

Abdominal pain is an important clinical symptom of pancreatic cancer (PC) that is characterized by intermittent or persistent pain and contributes to a poor quality of life in PC patients [1, 2]. Some patients present with abdominal pain, particularly in the early stages of the disease, but most patients experience pain in advanced stages [3]. At present, the lack of comprehensive research addressing the pain mechanism has hampered therapies.

There is increasing evidence that PC pain is triggered by pancreatic neuropathy [4]. Recent studies show transient receptor potential vanilloid 1 (TRPV1), a nonselective cation channel with a preference for calcium, is involved in pancreatic pain [5–7]. Indirect evidence suggests neuropeptides, such as calcitonin gene-related peptide (CGRP) and substance P (SP), play a key role in pain generation and that the suppression of CGRP and SP could reduce pain [8, 9]. The activation of TRPV1 in neurons induces the secretion of SP and CGRP and induces neuropathic pain in the neurons of the pancreas or dorsal root ganglia (DRG) [10, 11]. Moreover, in a study on pancreatitis, quantified pain by stimulating the abdomen with von Frey hairs, elevated expression of SP and CGRP was observed in DRG [12]. Thus, TRPV1 and the pain-relative factors may share more interactions in the process of PC pain.

The neurotrophic factors are known to have a role in the biological behavior of these cells [13]. The neurotrophic factor family consists of the glial cell line-derived neurotrophic factor (GDNF) family and four additional members: nerve growth factor (NGF), brain-derived neurotrophic factor (BDNF), neurotrophin 3 (NTF3), and NTF4 [14, 15]. Research about chronic pancreatitis suggested NGF and their receptors were involved in pain generation [16]. In addition, neurturin (NRTN), a member of the GDNF family, contributes to neuropathic pain and neuronal plasticity in PC [17].

The sonic hedgehog (sHH) signaling pathway, a major regulator of cell proliferation and differentiation [18], contributes to PC metastasis and the development of pancreatic fibrosis [19, 20]. The binding of sHH to Patched (PTCH) allows SMO (Smoothened) to activate downstream factors, such as Gli1 transcription factors (primarily Gli2), and regulate target gene expression [21, 22].

As reported previously, sHH regulates nociceptive sensitization and guides the spatial pathfinding of raphespinal tract axons [23, 24]. In addition, sHH signaling activates pancreatic stellate cells (PSCs) [25–27]. More recently, the focus of PC research has shifted to the effect of PSCs, which produce the pancreatic tumor stroma, on PC progression [28]. There is abundant evidence suggesting that PSCs affect PC development [29, 30]. However, few studies address the interactions between PC, neural components and PSCs in the generation of PC pain; we reasoned that the sHH pathway may provide insight on the pain generation mechanism. Therefore, in this study, we take the integrated analysis to demonstrate that PC pain originates from the sHH signaling pathway, which is activated in PSCs by sHH secreted from PC cells, and that the neurotrophic factors derived from PSCs mediate the pain mechanism by regulating the expression of TRPV1, SP, and CGRP.

## RESULTS

### Expression of TRPV1, SP, and CGRP in DRG neurons

**Figure 1:**
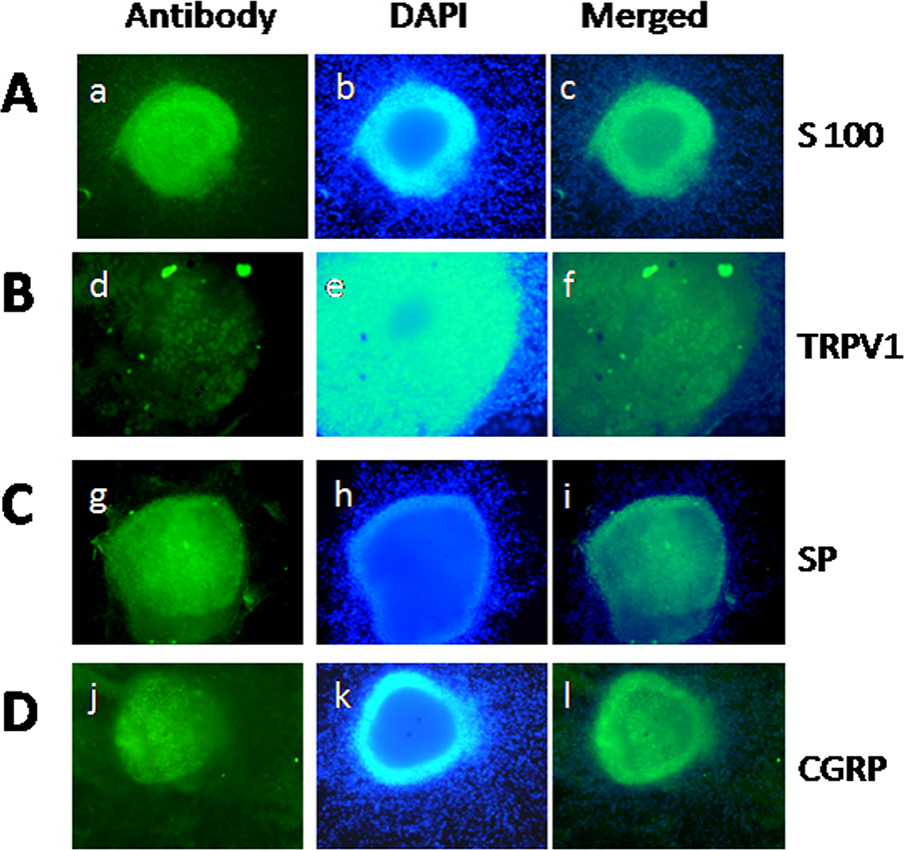
Qualification of DRG and staining of TRPV1, SP, and CGRP in DRG neurons (**A**) Identification of DRG extracted from newborn rat by S100 staining. (**B–D**) Staining of TRPV1, SP, and CGRP in DRG neurons. (a, d, g, j) S100, TRPV1, SP, and CGRP-positive neurons (green) in DRG, respectively. (b, e, h, k) Nuclear staining with DAPI. (c, f, i, l) Merge of staining is shown.

To investigate the possible relationship between PC and pain *in vitro*, we extracted DRG from newborn rats [31, 32]. The neurons of DRG were identified by the immunofluorescent staining of S100, which is a characteristic protein for nerves (Figure 1A). Moreover, we detected TRPV1, SP, and CGRP expression in nerve fibers within DRG (Figure 1B, 1C, and 1D). The co-expression of TRPV1 and these pain factors in DRG provided the basis and feasibility for our subsequent PC pain research.

### PSCs increase the expression of TRPV1 and pain factors within rat DRG in a co-culture system

To determine whether PSCs mediate pancreatic pain in PC, we first designed four different culture systems for PC cells, PSCs, and DRG. To eliminate interference from intercellular interactions in these systems, these co-culture systems were not mixed directly, but an indirect co-culture via culture medium exchange was adopted. The culture models are shown in Supplementary Figure S1. In addition, PSCs were extracted, separated, and cultured from cancer tissues of PC patients 7 days before the experiment.

**Figure 2:**
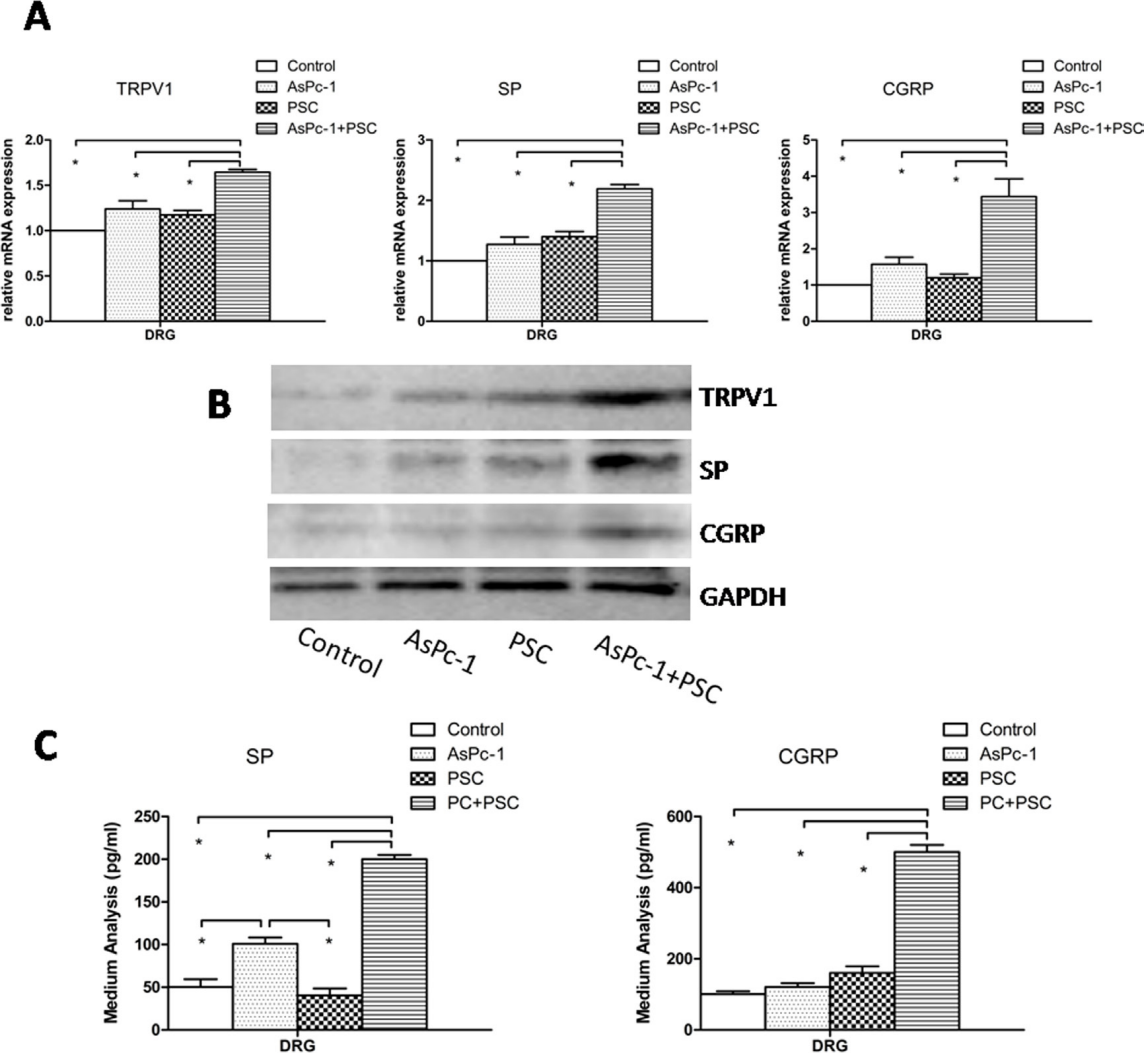
Expression and secretion of TRPV1, SP, and CGRP in DRG (**A**) The graph shows the mRNA expression of TRPV1, SP, and CGRP in DRG in different culture systems. The expression levels in co-culture of AsPc-1 cells with PSCs were higher than in other groups (*p* < 0.05) or in DRG cultured alone as a control. (**B**) The protein expression of TRPV1, SP, and CGRP in DRG in different culture systems determined by Western blotting. The protein results were similar to mRNA; DRG were cultured alone as a control. (**C**) Co-culture of AsPc-1 cells and PSCs increased the secretion of pain factors, SP, and CGRP by ELISA, compared with other groups (*p* < 0.05).

The mRNA results showed the expression levels of TRPV1, SP, and CGRP in DRG were much higher in the co-culture of PC cells and PSCs than in monocultures (PC cells or PSCs) or in the control group (Figure 2A) (*p* < 0.05). Additionally, there were no significant differences between the other three culture groups (control, PC cells, and PSCs). To supplement the mRNA analyses, Western blotting was used to quantify protein levels, and the results showed the protein expression was similar to mRNA expression (Figure 2B). As expected, no differences were observed among the control, PC, and PSC groups in terms of protein expression levels.

Based on the hypothesis that pain generation is due to the secretion of pain factors, we analyzed the concentrations of SP and CGRP in the culture medium of DRG by ELISA. The results show the secretion of pain factors is much greater in the co-culture group (Figure 2C) (*p* < 0.05). Thus, the high expression and secretion of TRPV1 and pain factors from DRG induced by PSCs in the co-culture system indicates that PSCs play a key role in PC pain.

### The sHH signaling pathway is involved in the regulation of TRPV1 and pain factors in the co-culture system

**Figure 3:**
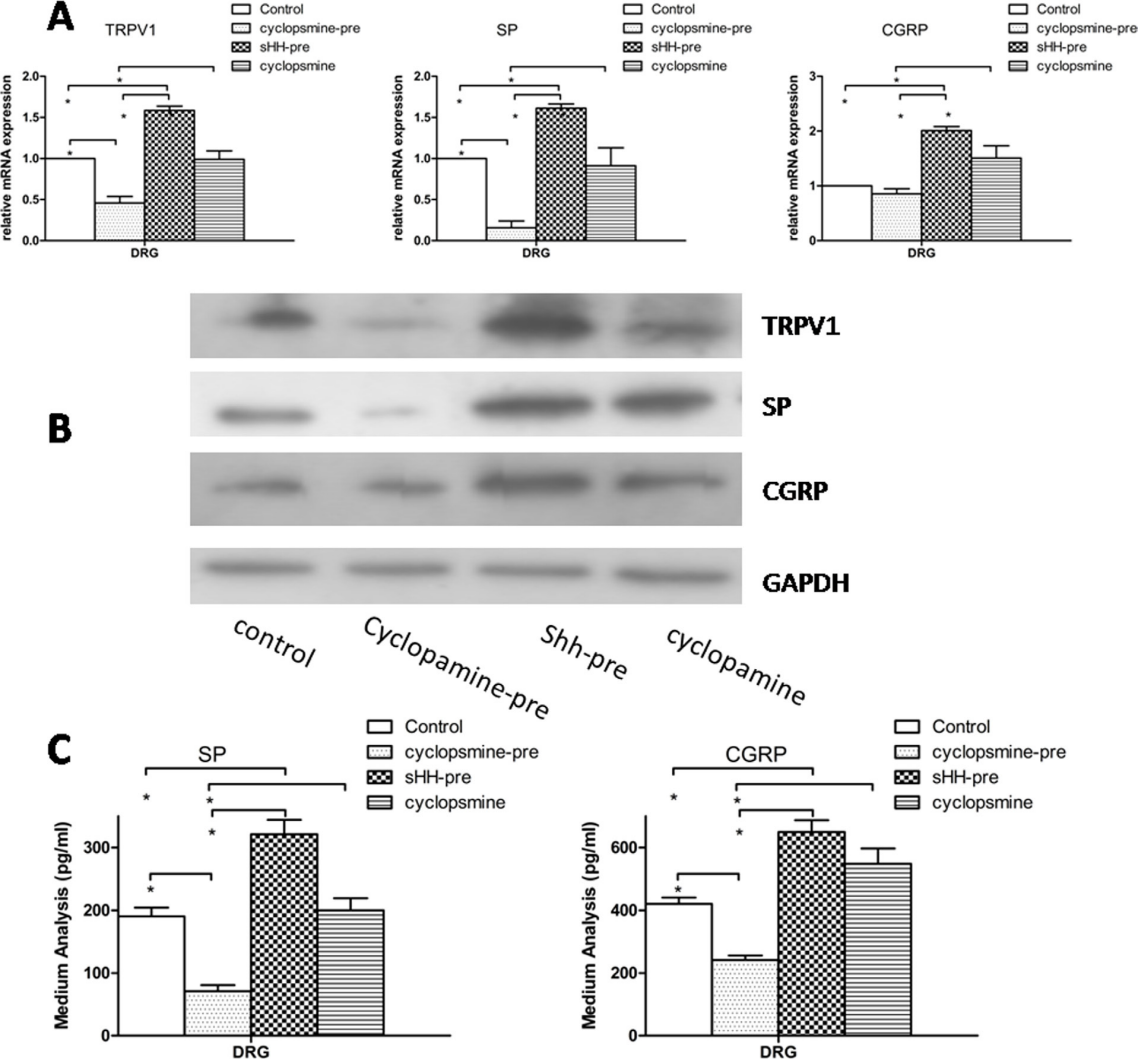
Effect of sHH and cyclopamine on expression and secretion of TRPV1, SP and CGRP in DRG in co-culture system (**A**) The cyclopamine-pre group (cyclopamine was used to treat the co-culture of PC cells and PSCs before removing the medium (1 ml) and adding it to DRG) suppressed the mRNA expression of TRPV1, SP and CGRP compared with control and other groups (*p* < 0.05). However, the sHH-pre group (sHH was used to treat the co-culture of PC cells and PSCs before removing the medium (1 ml) and adding it to DRG) increased the mRNA expression compared to control or the cyclopamine-pre group (*p* < 0.05). In contrast, the cyclopamine group (cyclopamine was used to treat DRG directly) was not different compared to control. (**B**) The Western blotting results were similar to mRNA results. (**C**) Cyclopamine-pre decreased the secretion of SP and CGRP compared with control and sHH-pre; in contrast, sHH-pre enhanced the secretion compared to other groups (*p* < 0.05). No effect of cyclopamine on the secretion of SP and CGRP was observed (*p* > 0.05).

To confirm the function of the sHH signaling pathway in our co-culture system, we examined the effects of recombinant sHH and cyclopamine on the expression and secretion of TRPV1 and pain factors. The co-culture system composed of PC cells (AsPc-1), PSCs, and DRG is shown in Supplementary Figure S1D. The results showed the expression and secretion of pain factors was inhibited in the cyclopamine-pre group (added to the media of PSCs). However, cyclopamine added to DRG media did not produce similar results (*p* > 0.05). The expression and secretion of TRPV1, SP and CGRP were increased in sHH group compared with the control group (Figure 3) (*p* < 0.05). However, cyclopamine added to DRG media did not produce similar results (*p* > 0.05). This interesting finding suggests the sHH signaling pathway is functional in PC cells and PSCs but not in DRG. We also found that recombinant sHH-pre (added to the medium of PSCs) obviously increased the expression and secretion of these factors compared to control (*p* < 0.05).

We also measured sHH expression in different PC cell lines (Supplementary Figure S2). Panc-1 (low sHH expression) and AsPc-1 cells (high sHH expression) were used for transfection (Supplementary Figure S3). The results show that the sHH signaling pathway played an important role in regulation TRPV1, SP, and CGRP and was activated by sHH from PC cells.

### PC cells activate the sHH signaling pathway and increase the expression of NGF and BDNF in PSCs

**Figure 4:**
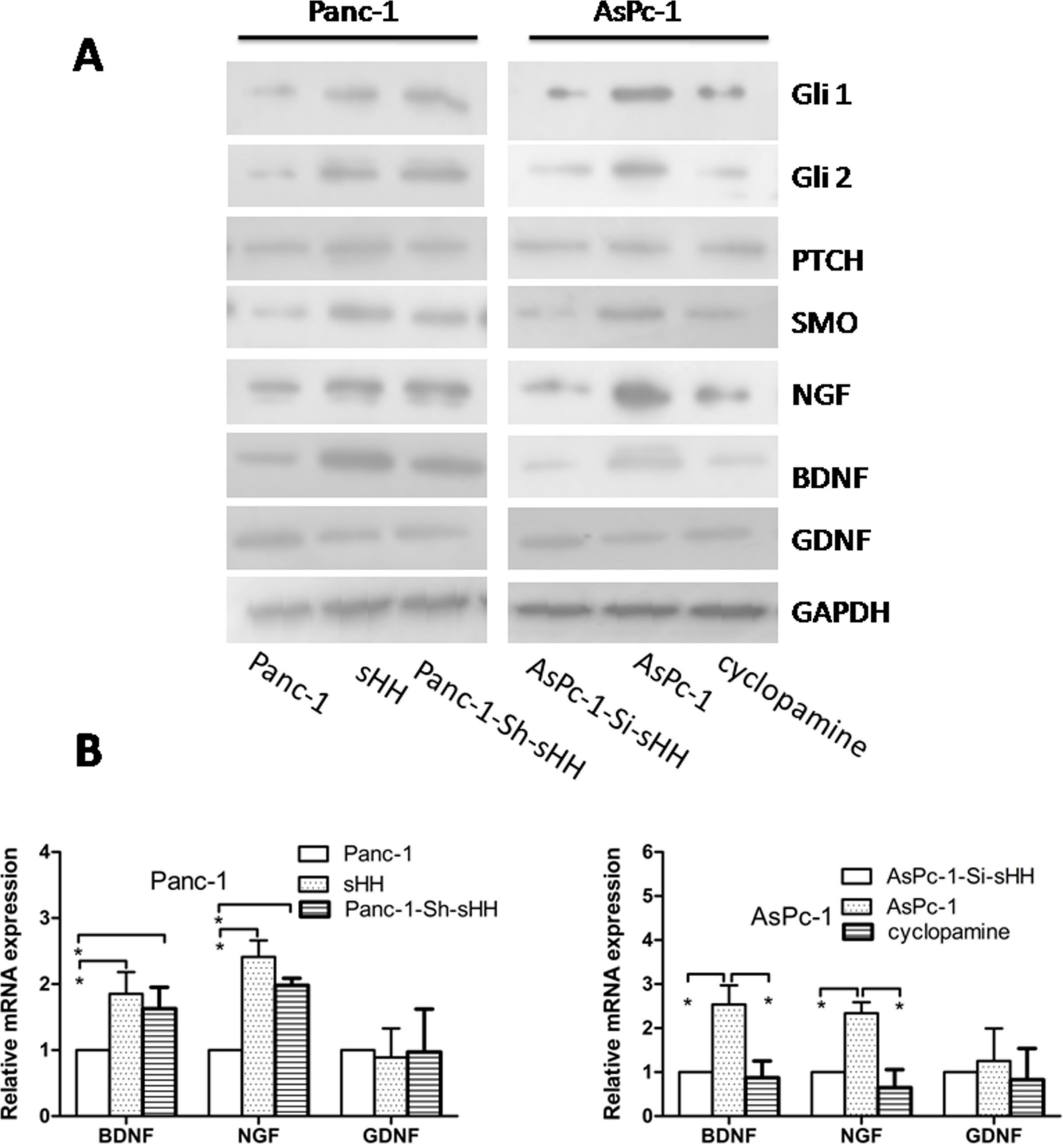
PC cells activate the sHH signaling pathway and increase expression of NGF and BDNF in PSCs (**A**) Panc-1-Sh-sHH cells and recombinant sHH up-regulated sHH signaling molecules (Gli1, Gli2 and SMO) and increased the expression of NGF and BDNF in PSCs compared with the Panc-1 cells group; furthermore, AsPc-1-Si-sHH and cyclopamine treatment reduced the expression of signal molecules (Gli1, Gli2 and SMO) and NGF and BDNF compared to the AsPc-1 cells group (*p* < 0.05). However, there was no difference in GDNF among the groups. (**B**) Panc-1-Sh-sHH cells and recombinant sHH increased the mRNA expression of neurotrophic factors; in contrast, AsPc-1-Si-sHH and cyclopamine reduced their expression (*p* < 0.05). However, the stimulation had no effect on GDNF for the two cell types. Panc-1-Sh-sHH: Panc-1 cells (low sHH expression) were stably transfected with sHH plasmids; AsPC-1-Si-sHH: AsPc-1 cells (high sHH expression) were transiently transfected with sHH siRNA.

To investigate whether sHH secretion from PC cells could activate the sHH signaling pathway in PSCs, we measured the expression of sHH pathway signaling molecules in PSCs in an indirect culture system (Supplementary Figure S1B) by Western blotting. Overexpressing sHH in Panc-1 cells induced the expression of the transcription factors Gli1 and Gli2 in PSCs compared with untransfected Panc-1 cells, and this induction was similar to that with positive control treated with recombinant sHH (Figure 4A). We also found knock down of sHH in AsPc-1 cells using siRNA inhibited the expression of these target molecules in PSCs (Figure 4A). Thus, these results showed the sHH signaling pathway in PSCs could be activated by sHH secretion from PC cells in an indirect co-culture system.

Considering that neurotrophic factors play important roles in pain and to confirm whether the activation of the sHH signaling pathway significantly contributes to the induction of neurotrophic factors in PSCs, we measured the expression of NGF, BDNF, and GDNF in PSCs using immunofluorescence (Supplementary Figure S4). We then demonstrated that NGF and BDNF were obviously increased in PSCs treated with media from PC cell lines that overexpress sHH, but this increased expression could be reversed by cyclopamine or siRNA against sHH (Figure 4A and 4B) (*p* < 0.05). We did not observe differential expression of GDNF in any group. Thus, the activation of the sHH signaling pathway induced the expression of NGF and BDNF in PSCs.

### Anti-NGF treatment results in suppression of TRPV1, SP, and CGRP expression within DRG in our co-culture system

**Figure 5:**
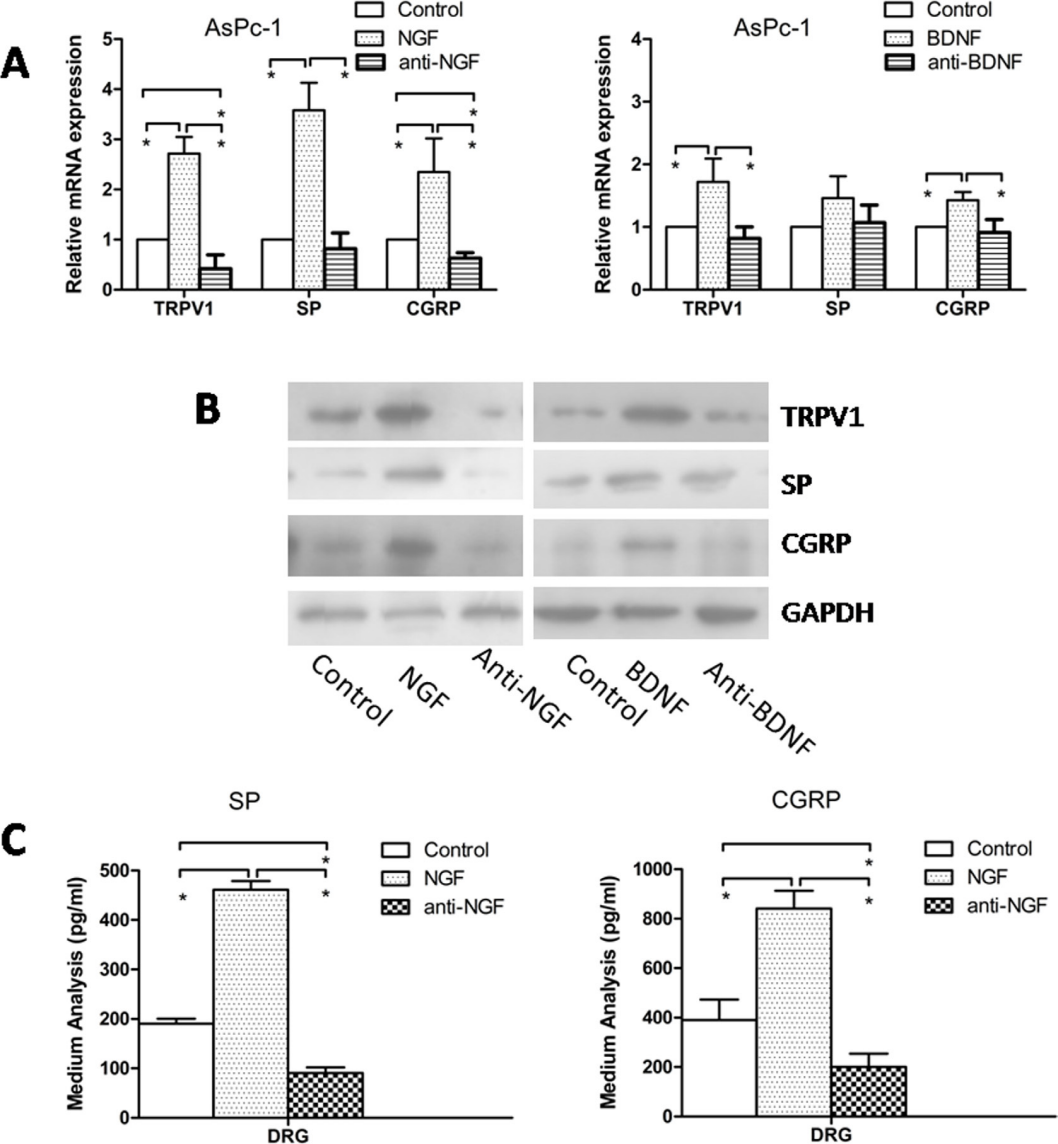
Effect of anti-NGF and anti-BDNF on expression of TRPV1, SP, and CGRP in DRG in co-culture system (**A**, **B**) Recombinant NGF or BDNF increased the mRNA and protein expression of TRPV1, SP and CGRP compared with control, and anti-NGF neutralizing antibody suppressed TRPV1, SP and CGRP expression compared to control. However, anti-GDNF had no effect on the expression of TRPV1, SP, and CGRP. (**C**) Recombinant NGF enhanced the secretion of SP and CGRP, and anti-NGF reduced the secretion (*p* < 0.05).

To determine whether the PSC-induced increased expression of TRPV1 and pain factors in DRG was mediated by neurotrophic factors, we quantified the expression of TRPV1, SP, and CGRP within DRG treated with neutralizing antibodies to NGF or BDNF in our co-culture system of AsPc-1 and PSCs (Supplementary Figure S1D). The antibody to NGF suppressed TRPV1, SP, and CGRP expression compared to control, and the expression levels were increased by recombinant NGF and BDNF added into the system (Figure 5A and 5B). However, the antibody to BDNF didn't decrease the expression of TRPV1, SP, and CGRP. To confirm the effect of NGF and anti-NGF on the secretion of SP and CGRP, an additional ELISA experiment was conducted. A similar result with PCR and western blotting was observed (Figure 5C). Recombinant BDNF also induced secretion, but anti-BDNF had no effect on the secretion of pain factors.

### sHH signaling pathway activation of PSCs results in potentiation of TRPV1 current in DRG in co-culture system

To determine whether PSCs and the sHH signaling pathway affect TRPV1 current, we chose AsPc-1 cells for our electrophysiology studies in a co-culture system (Supplementary Figure S1D).

**Figure 6:**
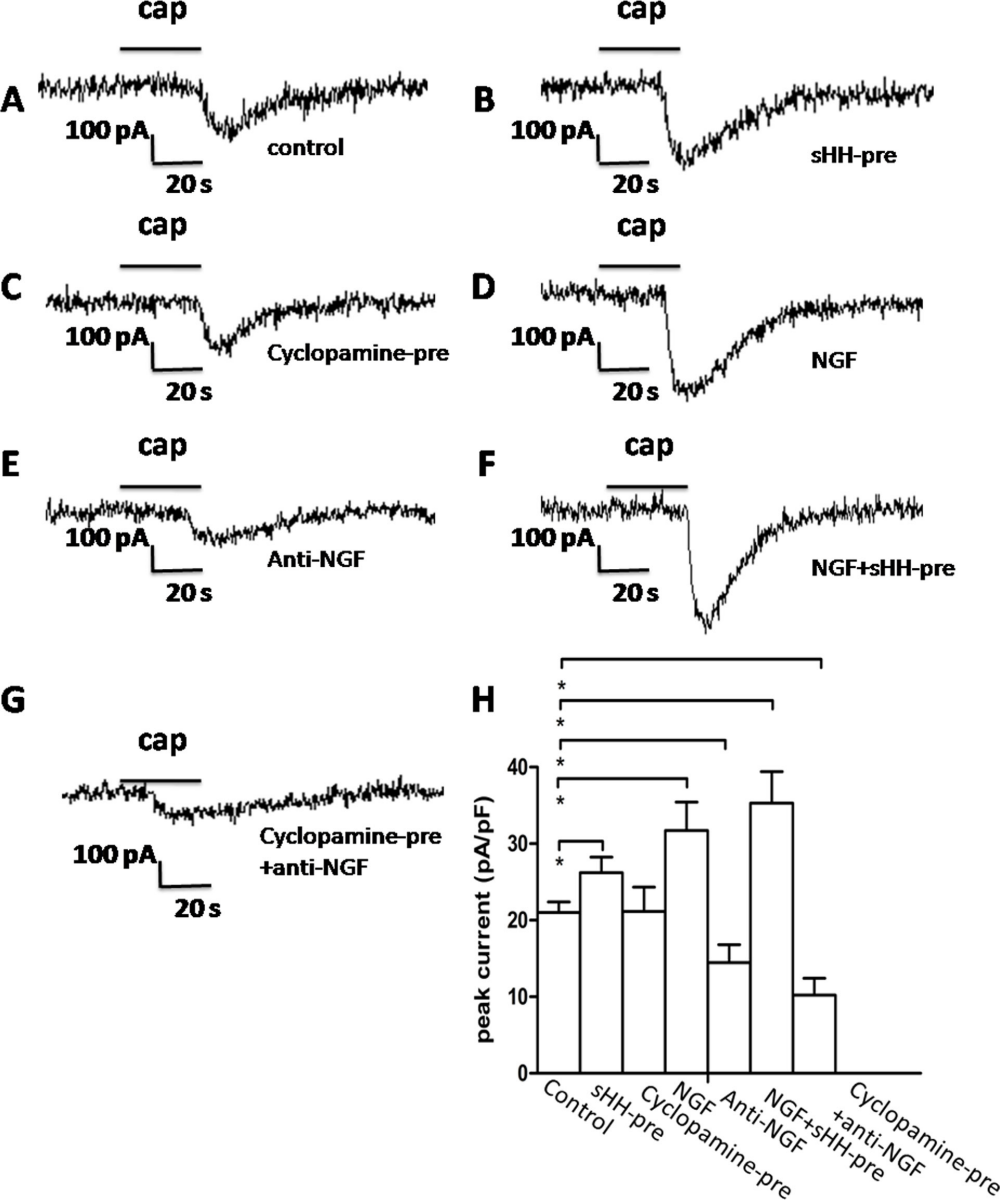
The sHH signaling pathway and NGF potentiated TRPV1 currents in DRG in co-culture system (**A**) Representative TRPV1 current evoked by capsaicin (1 μM) application to DRG neurons as a control. (**B**) TRPV1 current was potentiated by sHH-pre (used to treat the co-culture of PC cells and PSCs) in DRG neurons. DRG neurons were treated with the co-culture medium (1 ml) of PC cells and PSCs that was pretreated with sHH for 12 h before a 2 min application of capsaicin (1 μM) (*n* = 12 cells, *p* < 0.05). (**C**) Cyclopamine-pre treatment attenuated TRPV1 currents in DRG neurons treated with co-culture medium before the application of capsaicin (1 μM) (*n* = 17 cells, *p* < 0.05). (**D**) NGF enhanced TRPV1 currents in DRG neurons treated with NGF before a 2 min application of capsaicin (1 μM) (*n* = 15 cells, *p* < 0.05). (**E**) Anti-NGF treatment attenuated TRPV1 currents (*n* = 20 cells, *p* < 0.05). (**F**) Treatment with combined sHH-pre and NGF obviously increased TRPV1 currents (*n* = 11 cells, *p* < 0.05). (**G**) Treatments of cyclopamine-pre and anti-NGF significantly inhibited the currents (*n* = 22 cells, *p* < 0.05). (**H**) The bar graph shows the mean peak current above shown in DRG neurons compared with the control group.

TRPV1 currents were potentiated by the perfusion of recombinant sHH, NGF, or their combination (Figure 6B, 6D and 6F); the application of capsaicin (cap, 1 mM) as a control elicited a depolarizing in ward current (Figure 6A) (*p* < 0.05). Unfortunately, the cyclopamine-pre group didn't have any obvious difference compared with control (Figure 6C). Anti-NGF treatment was associated with a significant reduction of DRG current (Figure 6E) (*p* < 0.05). In addition, decreased current was observed in the both the cyclopamine and anti-NGF perfusion groups compared with the control group (Figure 6G).

### Antagonism of TRPV1 diminishes SP and CGRP expression and secretion in DRG in the co-culture system

To determine whether TRPV1 contributed to the expression and secretion of SP and CGRP in our experiment, AsPc-1 cells were chosen for the co-culture system (Supplementary Figure S1D). We quantified the expression levels of SP and CGRP in DRG and in the medium by Western blotting and ELISA. The results showed capsazepine, an inhibitor of TRPV1, alone or combined with cyclopamine-pre and/or anti-NGF obviously diminished the expression and secretion of SP and CGRP compared with the control (Supplementary Figure S5) (*p* < 0.05). Of note, the inhibitory effect of the combined groups was stronger than the effect from treatment with capsazepine alone. Additionally, the greatest inhibitory action was observed in the group with the three inhibitors combined. However, there was no difference among the cyclopamine-pre, anti-NGF, and capsazepine groups.

### sHH signaling pathway activation of PSCs results in sensitization to pain; anti-NGF could reduce the pain-related behavior in a nude mouse model

**Figure 7:**
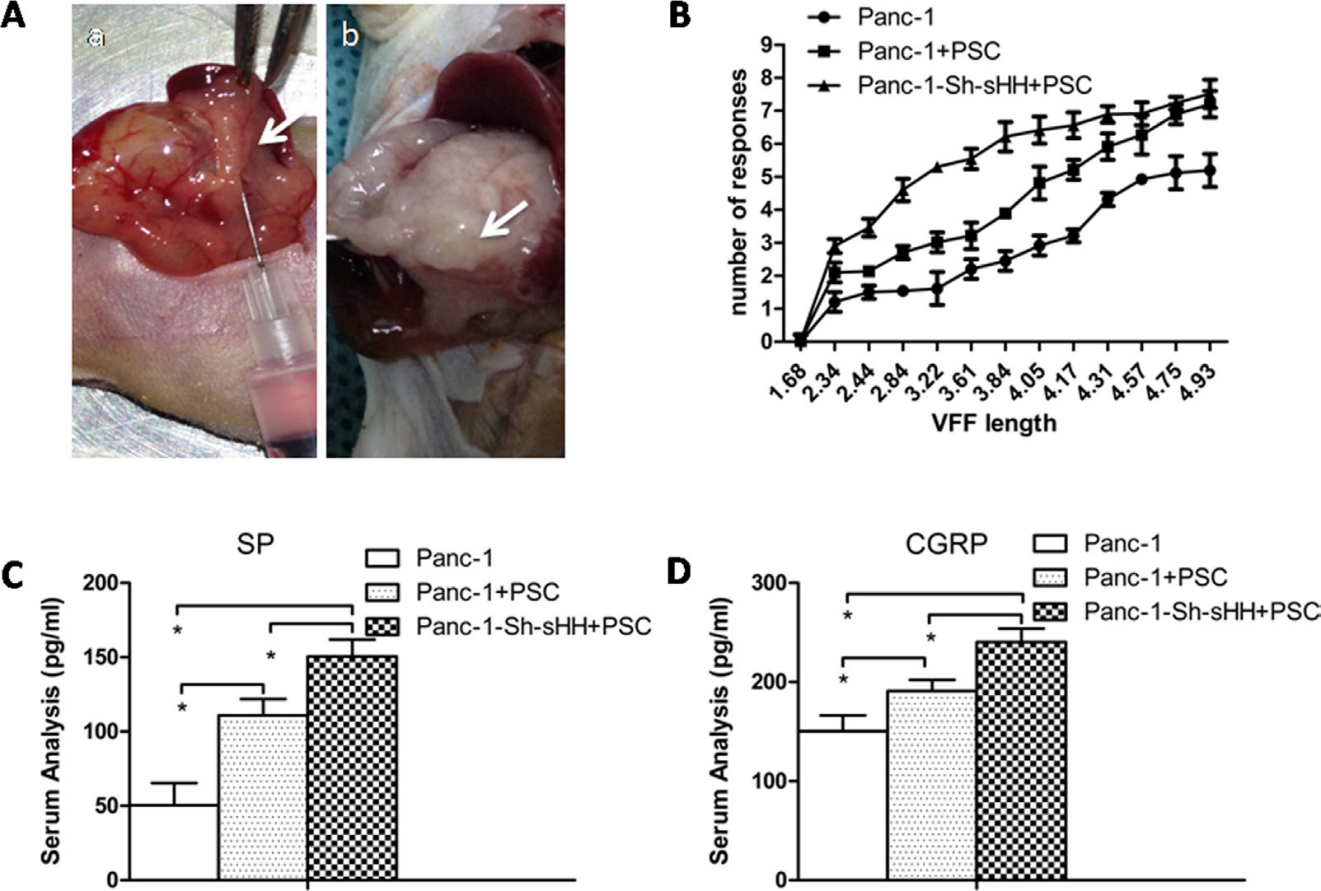
PSCs aggravated pain behaviors and induced secretion of SP and CGRP in mice (**A**) Panc-1 cells or mixed Panc-1 cells and PSCs were co-injected into the pancreases of nude mice (a). After 4 weeks, tumors were apparent (b). (**B**) The graphs show the average number of responses to mechanical stimulation 4 weeks after inoculation. The response rate was greater in the co-injection of Panc-1 cells and PSCs compared with control (*p* < 0.05). In contrast, the co-injection of Panc-1-Sh-sHH cells and PSCs had the strongest effect on pain behaviors. (**C**, **D**) The secretion of SP and CGRP into serum gradually increased in the following order: PC cells grou*p* < mixed PC cells and PSCs group < mixed Panc-1-Sh-sHH and PSCs group.

From our *in vitro* studies, we concluded that PSCs and activation of the sHH signaling pathway played a key role in the regulation of TRPV1 and pain factors (SP and CGRP). Additionally, we found neurotrophic factors were involved in the mechanism. To determine whether PSCs mediate pancreatic pain behavior in a mouse model, we designed an *in situ* tumor model in nude mice (Figure 7A). Panc-1 cells or a mixture of Panc-1 cells and PSCs were injected into the pancreases of nude mice. After 4 weeks, we measured the sensitivity of the abdomen to mechanical stimulation by the von Frey filament probing method in three different *in situ* tumor models (Figure 7B). The frequency of response to mechanical stimulation for mice injected with mixed Panc-1 cells and PSCs was greater than that for mice injected with PC cells alone (Figure 7B) (*n* = 8) (*p* < 0.05). The group of Panc-1-Sh-sHH combined with PSCs produced the strongest sensitivity of the three groups (Figure 7B) (*n* = 8) (*p* < 0.05). Subsequently, we quantified the secretion of SP and CGRP in the sera of each group of mice 2 hours after the mechanical stimulation. The results showed the concentrations of these pain factors gradually increased in the following order: Panc-1 cells group < PC cells and PSCs group < Panc-1-Sh-sHH cells and PSCs group (Figure 7C and 7D) (*n* = 8) (*p* < 0.05). Thus, PSCs and activation of the sHH signaling pathway are important for pancreatic pain.

In addition, to investigate the effect of NGF and TRPV1 on the tumor model, the mixture of AsPc-1 cells and PSCs was co-injected into the pancreases of mice. Results show that combining both inhibitors significantly suppressed the pain-related behavior more than using either inhibitor alone (Supplementary Figure S6).

## DISCUSSION

Abdominal pain is a common symptom in patients with PC. Traditional pain medications, including analgesics and opiates, and operations such as celiac plexus blocks [33] are only partially effective in controlling PC pain.

There are many reasons for pain generation, e.g., the damage of the neuronal sheath, inflammatory factors, and cell signaling pathways between PC cells and nerves [34]. However, the specific and exact mechanism contributing to pain generation in PC is unclear. Here, we attempt to provide some understanding by studying the relationship of PSCs, the sHH signaling pathway and neurotrophic factors.

PSCs are a part of the pancreatic stroma. There is now compelling evidence that PSCs interact with PC cells, immune cells, and neuronal cells to promote cancer progression. The influence of PSCs on novel therapeutic approaches is becoming a hot target for researchers [35, 36]. In this article, we first described the role of PSCs in the co-culture system. The results showed that the expression levels of TRPV1, SP, and CGRP in DRG were much higher in the co-culture system. Furthermore, the secretion of SP and CGRP was increased. Thus, the results implied PSCs are necessary in the co-culture system and may play an important role in PC pain generation.

In addition, it needs to be emphasized that the DRG was regarded as a whole; we did not differentiate constituents, such as nerve cells, gliocytes or schwann cells, except when we used nerve cells in the electrophysiological recordings. Furthermore, to clarify the source of sHH and the activation of the signaling pathway, the indirect co-culture of PC cells and PSCs was used. All the co-culture systems in this study involving co-culture of PC cells, PSCs and DRG are the first of their kind to be used in PC pain research, suggesting that these systems are imperfect and could be improved in further study. However, the advantage of our system is that it focuses on the function of PSCs and DRG while removing interference and obstacles caused by other cells in the microenvironment.

It has been shown that sHH plays a functionally important role in the development of PC [37]. In this study, we investigated the contribution of sHH to PC pain by detecting the expression levels of TRPV1, SP, and CGRP in DRG after treatments with recombinant sHH and cyclopamine in the co-culture system. Thus, we showed that the sHH signaling pathway was involved in the regulation of PC pain *in vitro* and that the sHH signaling pathway was activated in PSCs. However, when cyclopamine was added into the medium of DRG, the results were not statistically significant compared with the control group, suggesting that, at least in this system, the sHH signaling pathway was not activated in DRG.

The transfections results showed the expression of TRPV1, pain factors and the transcription factors Gli1 and Gli2 in PSCs were affected by sHH secreted from PC cells. These showed that the sHH signaling pathway was important for PSCs to elicit PC pain in the co-culture system. Some research has shown NGF can modulate the expression and function of TRPV1 [6]. We observed the expression of the neurotrophic factors NGF, BDNF, and GDNF was induced by the sHH pathway in PSCs. These results suggested that PC cells activated the sHH signaling pathway and increased the expression of NGF and BDNF in PSCs but had in significant effects on GDNF expression, suggesting that, at least at the level released from PC cells, sHH only affects NGF and BDNF expression.

To determine whether the PSC-induced expression of TRPV1 and pain factors are mediated by neurotrophic factors, the expression of TRPV1, SP, and CGRP within DRG was detected after treatment with neutralizing antibodies against NGF or BDNF in the co-culture system. We found decreased expression of TRPV1, SP, and CGRP in the anti-NGF group. Their expression increased after stimulation with recombinant NGF. In further experiments, anti-BDNF did not produce a similar result as anti-NGF treatment; however, recombinant BDNF enhanced the expression of TRPV1, SP, and CGRP. In agreement with the results of the above study, NGF is more significantly involved than BDNF in the mechanism of PSCs aggravating PC pain in our co-culture system. These data are in agreement with other studies [6, 17]. Further study was dedicated to NGF due to its apparently greater relative importance to the PC pain mechanism.

NGF is known to activate and promote the growth of sensory neurons. NGF binding with its receptor can alter the responsiveness of ion channels including TRPV1 [38, 39]. In this study, we focused on TRPV1, a key molecule for nociceptor responsiveness and excitability leading to the depolarization of the membrane and generate an action potential. We showed an obvious increase in the expression levels of TRPV1 in DRG, and we found that the sHH pathway and PSCs could sensitize sensory neurons through the activation of TRPV1 in our co-culture system. Furthermore, we found that anti-NGF could inhibit the TRPV1 current, whereas cyclopamine did not inhibit the current. Thus, our results showed the sHH pathway and PSCs were involved in the mechanism of PC pain in our co-culture system; moreover, the PSCs made a greater contribution by expressing NGF. These findings supplement data on the function of the sHH pathway and PSCs in previous research [6, 24, 28]. TRPV1 is only one of the many targets for pain receptors.

We have previously shown the role of PSCs and the sHH pathway in our *in vitro* co-culture system. Although some studies have found that TRPV1 is essential for pain in pancreatic disease [7, 40], we investigated the contribution of TRPV1 to the expression and secretion of SP and CGRP. Subsequently, we found that the inhibitory activity of capsazepine was potentiated in combination with cyclopamine and/or anti-NGF. Thus, together, our results suggest TRPV1 plays a strategic role in the PC pain mechanism. The combined application of these inhibitors further demonstrated the increasing TRPV1 activity. All the above data are cytological studies concerning PC pain, providing new theoretical evidence and research methods for studying the mechanism of PC pain.

In this study, we describe a pivotal role of the SHH pathway and PSCs in the pathogenesis of pain in PC only in an *in vitro* co-culture system. Then orthotopic tumor models were examined to confirm the experimental hypothesis. First, we co-injected the PC cells and/or PSCs into the pancreases of nude mice. Von Frey filament testing showed a significant increase of pain behavior in mice with the PSCs, and there was more secretion of SP and CGRP than in the control group. In contrast, there was an enhanced effect in sHH overexpressing cells on the pain behavior and secretion of pain factors. These results demonstrated sHH and PSCs were involved in the pain generation in animals, consistent with our previous studies *in vitro*. It is worth noting that the method of co-injection into the pancreas is the first of its kind in pain research and thus may have some unavoidable imperfections; however, we have demonstrated the feasibility in our preliminary experiment.

In addition, we investigated the effect of NGF and TRPV1 on the tumor model. Results suggested that the pain-related behaviors were suppressed after treatment with anti-NGF or capsazepine compared with co-injected untreated cells. Thus, our studies, both *in vivo* and *in vitro*, showed that NGF decreased the pain behaviors via effects on TRPV1. However, the mechanism of neuropathic pain is complex [2, 41], and other elements may be involved in the process. Thus, the results only illuminate a path for further study.

In conclusion, we have shown that PSCs play an essential role in the pain mechanism in PC. sHH secreted from PC cells activates the sHH signaling pathway in PSCs and enables the complex pain process via NGF and BDNF up-regulation or sensitization to TRPV1 and pain factors in sensory nerves. Our results should be regarded as one of the major potential mechanisms contributing to neuropathic pain in PC, and they represent a critical step forward for PC pain research.

## MATERIALS AND METHODS

### Cell culture and reagents

The human PC cell lines AsPc-1 and Panc-1 were obtained from the American Type Culture Collection (Manassas, VA) and cultured in DMEM supplemented with 10% fetal bovine serum (FBS) and 1% antibiotic/antimycotic in a humidified 5% CO_2_ atmosphere at 37°C. Antibodies against SHH, SMO, PTCH, Gli1, Gli2, NGF, BDNF, GDNF, TRPV1, SP and CGRP were purchased from Abcam, USA. Recombinant sonic hedgehog, NGF and BDNF were obtained from R & D Systems (Minneapolis, MN). Capsaicin was purchased from Sigma, USA.

### Real-time PCR and western blotting analysis

Total RNA was extracted using TRIzol (Invitrogen, CA, USA), and cDNA was synthesized using a Prime Script RT reagent Kit (TaKaRa, Dalian, China). The primers used for SYBR Green RT-qPCR are shown in Supplementary Table S1. The details are shown in Supplementary Methods.

### Plasmid construction and transfections

Cells seeded into small dishes were transfected with 100 nM siRNA using Lipofectamine RNAi MAX Reagent (Invitrogen, CA, USA) according to the manufacturer's instructions. The cells were used for further experiments 24 h after transfection. In addition, plasmids for the overexpression of sHH were constructed according to manufacturer's instructions to study the activities of the sHH pathway in PSCs (Roche, Penzberg, Germany).

### Co-culture system

In our study, different culture systems were designed to clarify the relationship among sHH, PSCs and pain factors. PC cells, PSCs and DRG were cultured together directly and indirectly. The culture medium was used to convey soluble factors. A model of the co-culture system is shown in Supplementary Figure S1.

### ELISA

To quantify the relative levels of neurotrophic and pain factors in culture media and serum, an enzyme-linked immunosorbent assay (ELISA) kit (R & D, Minneapolis, MN, USA) was used according to the manufacturer's instructions.

### Immunofluorescence

TRPV1, SP, and CGRP were localized to DRG, and NGF, BDNF, and GDNF were localized in PSCs by immunofluorescence. The details were shown in Supplementary Methods.

### Isolation and culture of mouse DRG and PSCs

The DRG were isolated from the newborn rat [42], stored on ice in DMEM:F12 (1:1) containing 20% FBS, and then seeded into 24-well plates. The co-culture of PC cells, PSCs and DRG was performed according to the study design. PSCs were prepared by the outgrowth method [43]. Fresh pancreatic tissue was minced and seeded in six-well plates on ice in DMEM:F12 (1:1). After culturing 4–5 days, cells grew out from the tissue clumps. The medium was changed every 3 days. All cells were maintained at 37°C in a humidified atmosphere of 5% CO_2_.

### Orthotopic transplantation tumor model in nude mice

Prepared PC cells and PSCs were injected or co-injected into the pancreases of nude mice exposed by midline laparotomy (4–6 sites; 20 μl total volume) (shown in Figure 7A). The groups included PC cells alone and a1:1 mixture of PC cells and PSCs (20 μl of cells at a total concentration of 1 × 10^6^/μl). After 4 weeks, the carcinoma model was highly successful (85%), and drug treatment and pain behavior could be tested.

### Von frey filament testing

Von Frey filament testing was performed as described previously [44]. After shaving the abdomens of nude mice, different intensities were used to stimulate the abdomen 20 times. Each stimulation lasted 2 seconds with an approximately 30-second interval between stimulations. The positive response consisted of lifting the belly and/or scratching or licking the abdomen. The number of responses was summed over all 20 stimulations [6].

### Electrophysiological recordings

Recordings of TRPV1 currents were obtained in DRG neurons using whole-cell patch-clamp techniques. The details were shown in Supplementary Methods.

### Drug treatments

*In vitro*, recombinant sHH was applied to PSCs or co-cultured PC cells and PSCs (labeled as sHH-pre) at 5 mg/ml for 12 h. As a control, cyclopamine, a sHH pathway inhibitor, was diluted to 18 μg/ml in PSC media (labeled as cyclopamine-pre) or DRG media(labeled as cyclopamine) and incubated with cells for 12 h. In addition, to detect the effect of neurotrophic factors, 60 ng/ml NGF or 50 ng/ml BDNF were added into the medium of DRG. As controls, 0.5 μg/ml polyclonal NGF antibody or 20 μg/ml BDNF antibody were used to block their effects in DRG media. Moreover, capsaicin (1 μM) was used to evoke TRPV1 currents in DRG. Furthermore, 20 μg/kg NGF antibody or 3 mg/kg capsazepine (TRPV1 inhibitor) were administered to animals by intraperitoneal administration.

### Statistics

The analyses of results were performed using the SPSS statistical software package (version 13.0). The significance of the data was determined using Student's *t* test or one-way analysis of variance (ANOVA) for *in vitro* and *in vivo* results. The of significance level was set at *p* < 0.05. Patch clamp data were analyzed by pClamp 9 (Axon Instrument, Foster city, CA). All results were expressed as the means ± SD. All the experiments have been repeated 3 times, *n* = 8 per group.

## SUPPLEMENTARY MATERIALS FIGURES AND TABLE



## Abbreviations

PC: pancreatic cancer
PSCs: pancreatic stellate cells
DRG: dorsal root ganglion
TRPV1: transient receptor potential vanilloid 1
CGRP: calcitonin gene-related peptide
SP: substance P
GDNF: glial cell line-derived neurotrophic factor
NGF: nerve growth factor
BDNF: brain-derived neurotrophic factor
NT3: neurotrophin 3
NRTN: neurturin
sHH: sonic hedgehog
PTCH: Patched
ELISA: enzyme-linked immunosorbent assay
