# Supplementary material for: Pancreatic stellate cells contribute pancreatic cancer pain via activation of sHH signaling pathway
Source: Oncotarget. 2016 Feb 27;7(14):18146–58. doi: 10.18632/oncotarget.7776 (PMC4951278; doi:10.18632/oncotarget.7776)
Supplement: Supplementary file 1 [file oncotarget-07-18146-s001.pdf]

Pancreatic stellate cells contribute pancreatic cancer pain via activation of sHH signaling pathway

Supplementary Materials

Real-time PCR

Total RNA was extracted using TRIzol (Invitrogen, CA, USA), and cDNA was synthesized using a Prime Script RT reagent Kit (TaKaRa, Dalian, China). The real-time experiments were conducted on an iQ5 Multicolor Real-Time PCR Detection System (Bio-Rad, Hercules, CA) using a SYBR Green Real-time PCR Master Mix (TaKaRa, CA, USA). The primers used for SYBR Green RT-qPCR are shown in Supplementary Table 1.

Western blotting analysis

Cells were extracted in lysis buffer [50 mM Tris (pH7.5), 150 mM NaCl, 1% NP 40, 0.5% sodium deoxycholate, 1 mM EDTA, and 0.1% SDS] containing a protease inhibitor cocktail (Sigma–Aldrich), and protein concentrations were measured with the DC Protein Assay (Bio-Rad Laboratories, Inc., Hercules, CA, USA). After separation on 7.5% SDS-polyacrylamide gels, proteins were transferred to nitrocellulose membranes (Amersham Bioscience), which were then incubated with primary antibodies at 4°C overnight. After washing 3 times, the membranes were incubated with horseradish peroxidase-conjugated secondary antibodies for 1 h. Immunoreactive bands were visualized using an enhanced chemiluminescence kit (Millipore, MA, USA).

Immunofluorescence

TRPV1, SP, and CGRP were localized to DRG, and NGF, BDNF, and GDNF were localized in PSCs by immunofluorescence. The prepared DRG and PSCs were washed 3 times with PBS and then fixed with 100 ml 4% paraformaldehyde in PBS. The cells were permeabilized in blocking buffer (0.1% Triton X-100 or 0.1–0.5% saponin, 10% NGS, 100 mM PBS, pH 7.4) for 1 hour at room temperature and then incubated with primary antibodies overnight at 4°C. The second day, cells were washed and incubated with FITC-conjugated goat anti-rabbit or anti-mouse IgG (1:500, Jackson ImmunoResearch, West Grove, PA) for 1 hour at room temperature.

Electrophysiological recordings

Recordings of TRPV1 currents were obtained in DRG neurons using whole-cell patch-clamp techniques. The details were shown in Supplementary methods Whole-cell voltage-clamp recordings were performed at RT(20–22°C) with an EPC-9 amplifier (HEKA Elektronik, Lambrecht/Pfalz, Germany). After DRG neurons were prepared, microelectrodes were pulled by a P97 puller (Sutter Instruments, Novato, CA, USA). The extracellular solution for patch clamping was Dulbecco's phosphate buffer containing 150 mM NaCl, 5 mM KCl, 2 mM $CaCl_2$, 1 mM $MgCl_2$, 10 mM HEPES, and 10 mM glucose, pH 7.4. The pipette solution contained 140 mM KCl, 5 mM NaCl, 1 mM $MgCl_2$, 0.5 mM $CaCl_2$, 5 mM EGTA, 3 mM Na_2-ATP, and 10 mM HEPES, pH 7.2. Data acquisition was controlled by Pulse and Pulse fit 8.5 software (HEKA Elektronik).

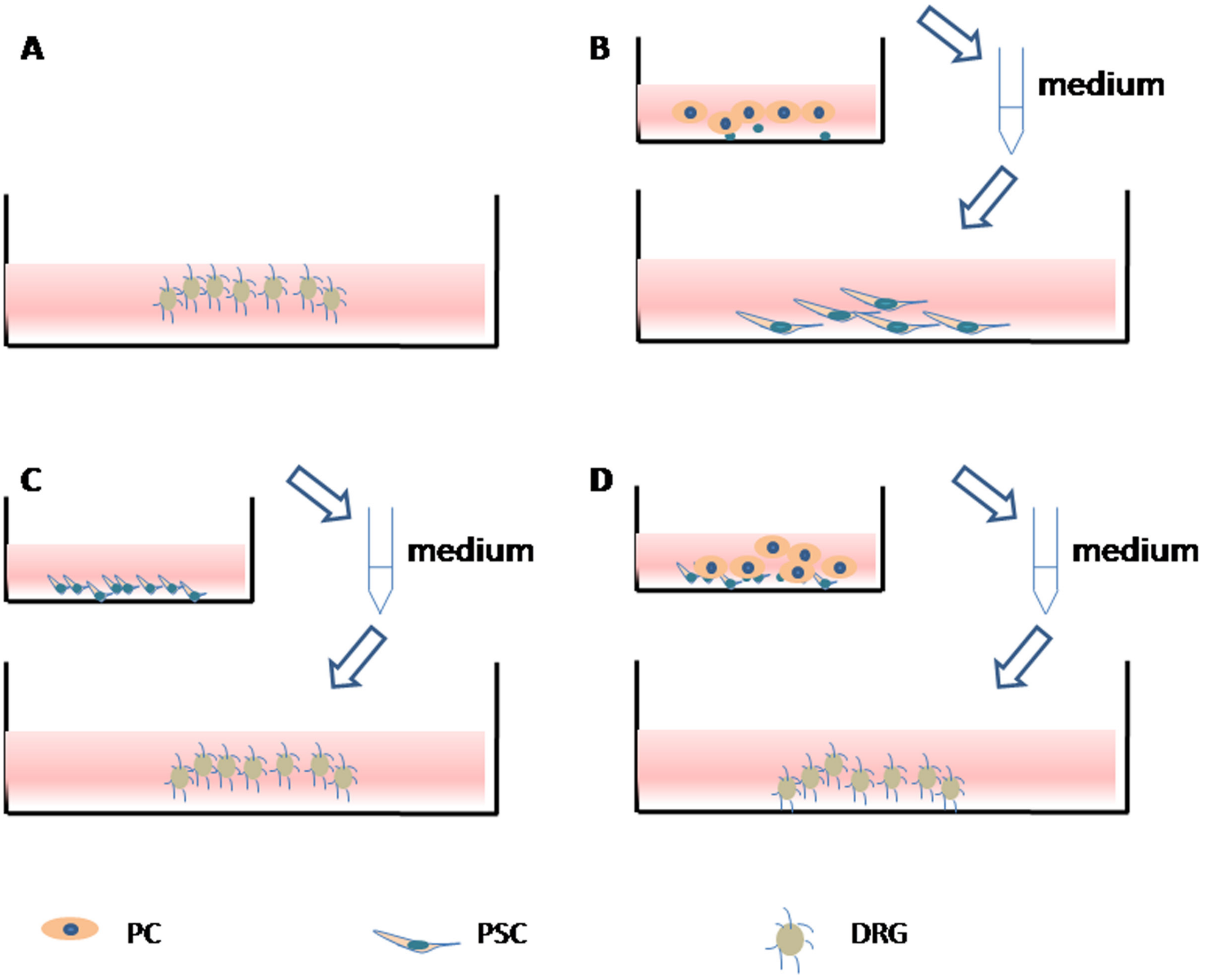

Supplementary Figure S1: Different co-culture system for PC cells, PSCs, and DRG. (**A**) DRG was cultured in DMEM/F12 (1:1) alone. (**B**) PSCs were indirectly co-cultured with PC cells. After PC cells and PSCs grew 24 h in 2 ml medium, 1 ml medium was removed from PSCs and replaced with 1 ml medium from PC cells. (**C**) PSCs were indirectly co-cultured with DRG. After PSCs and DRG grew 24 h in 2 ml medium, 1 ml DRG medium was removed and replaced with 1 ml of PSC medium. (**D**) DRG was indirectly co-cultured with PSCs and PC cells. After PSCs and DRG were cultured together for 24 h in 2 ml medium, 1 ml DRG medium was removed and replaced with 1 ml of co-cultured PC cells and PSCs medium.

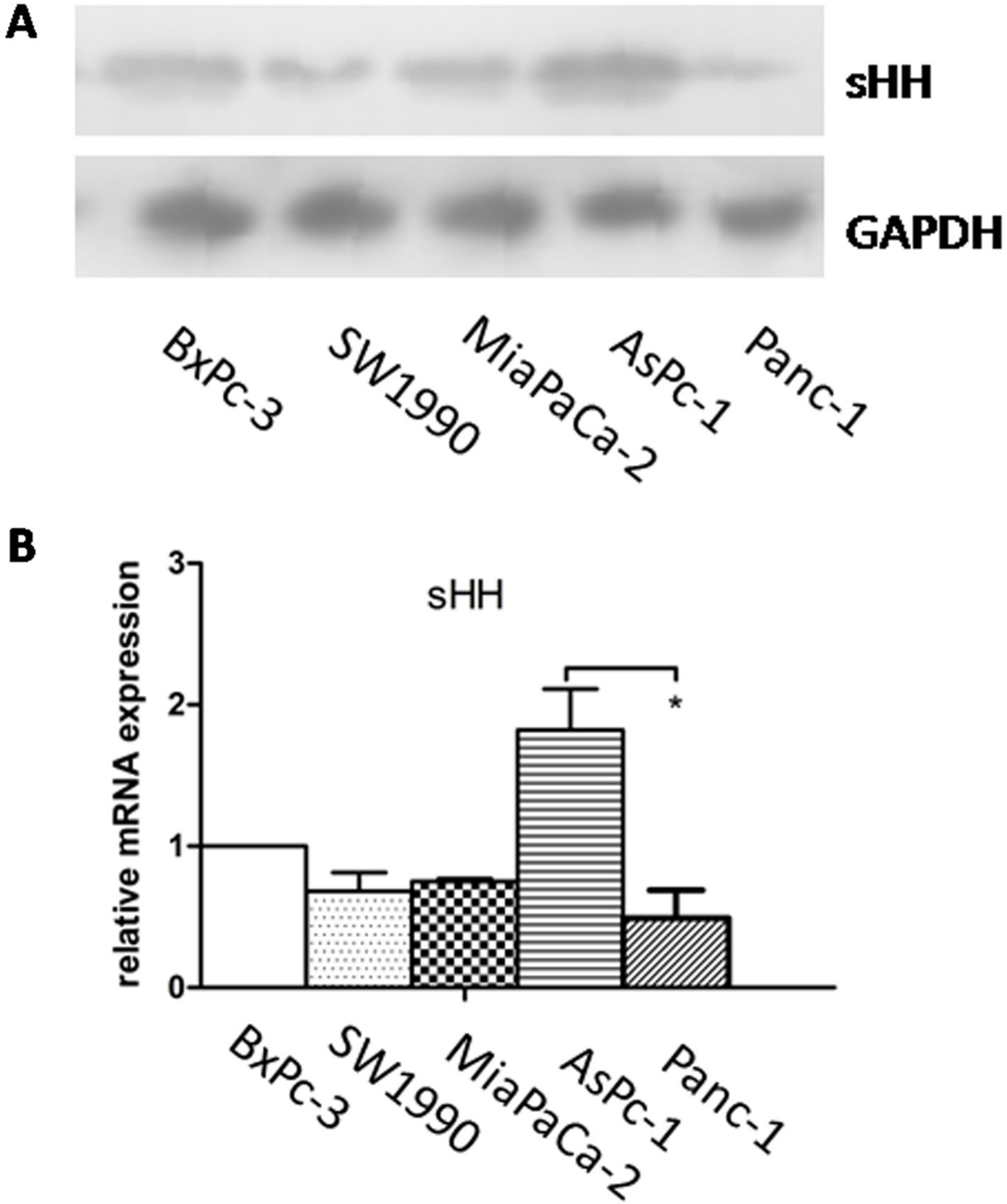

Supplementary Figure S2: PC cell linescreening for further study based on expression of sHH. (**A**) The protein expression of sHH in different PC cell lines. As seen, Panc-1 cells express low levels, and AsPc-1 cells express high levels of sHH. (**B**) The mRNA expression of sHH in different PC cell lines, which is similar to protein expression levels.

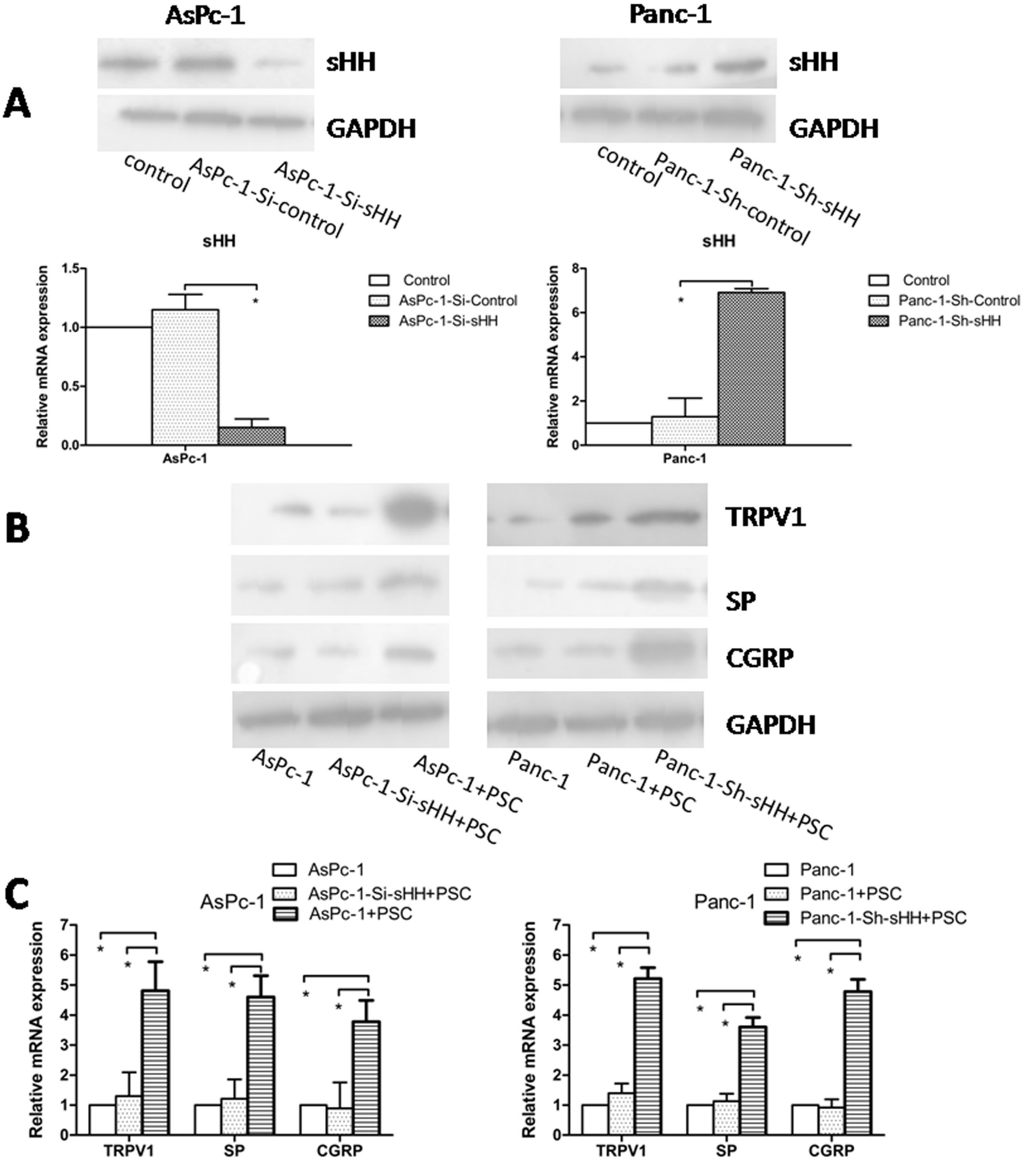

Supplementary Figure S3: The effect of sHH-transfected PC cells on expression of TRPV1, SP, and CGRP in DRG in co-culture system. (**A**) The transfection efficiencies were verified by real-time PCR and Western blotting. Panc-1 cells (low sHH expression) were transfected with sHH plasmids, and AsPc-1 cells (high sHH expression) were transfected with sHH siRNA. (**B**) The protein expression levels of TRPV1, SP and CGRP in the co-culture of AsPc-1-Si-sHH cells and PSCs were lower than those in the co-culture of AsPc-1 cells and PSCs. However, these levels were higher in co-cultured Panc-1-Sh-sHH cells and PSCs than in co-cultured Panc-1 cells and PSCs. (**C**) The mRNA expression changes of TRPV1, SP and CGRP in co-culture were similar to the protein results. ($p < 0.05$). Panc-1-Sh-sHH: Panc-1 cells (low sHH expression) were stably transfected with sHH plasmids; AsPC-1-Si-sHH: AsPc-1 cells (high sHH expression) were transiently transfected with sHH siRNA.

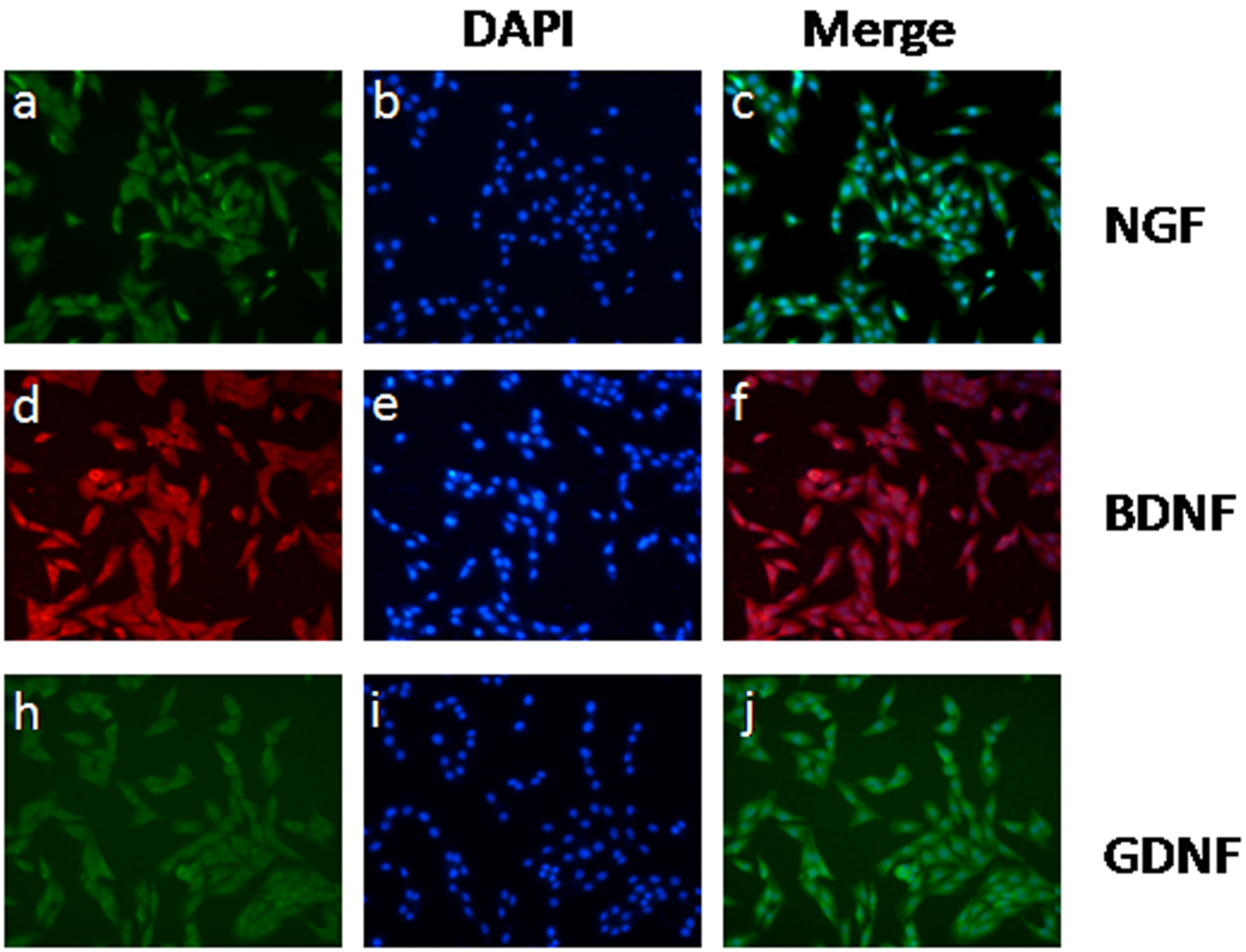

Supplementary Figure S4: Staining of NGF, BDNF, and GDNF in PSCs. (a,d,h) Expression of NGF (green), BDNF (red) and GDNF (green) in PSCs' cytoplasm, respectively. (b,e,i) Nuclear staining with DAPI. (c,f,j) Merge of staining is shown.

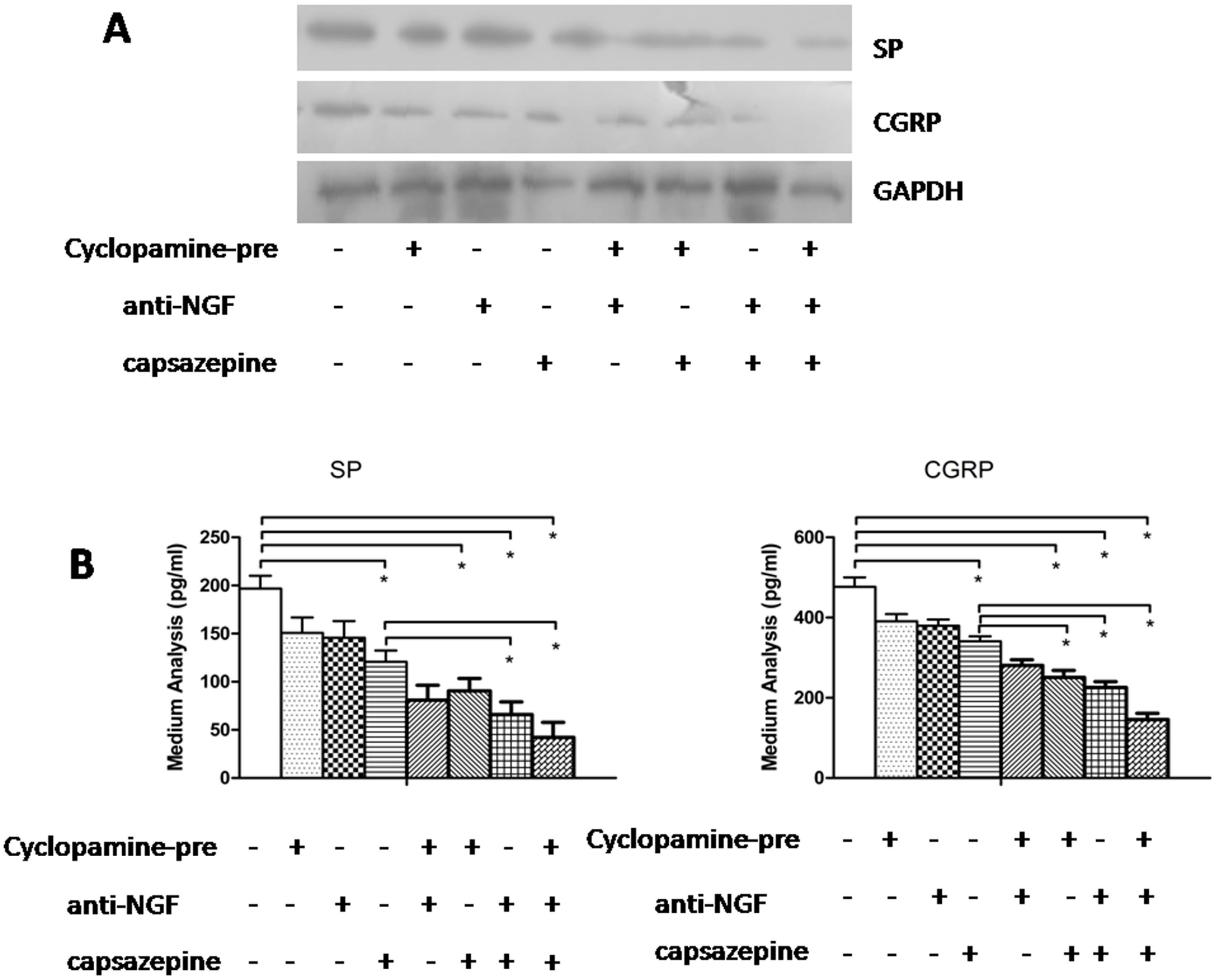

Supplementary Figure S5: Antagonism of TRPV1 diminished SP and CGRP expression and secretion in DRG in co-culture system. (**A**) Combined cyclopamine-pre and anti-NGF obviously reduced the expression of SP and CGRP compared with control or using either inhibitor alone ($p < 0.05$). (**B**) The graph shows that the secretion of SP and CGRP was lower in the combination group than in the control group or with either individual inhibitor ($p < 0.05$).

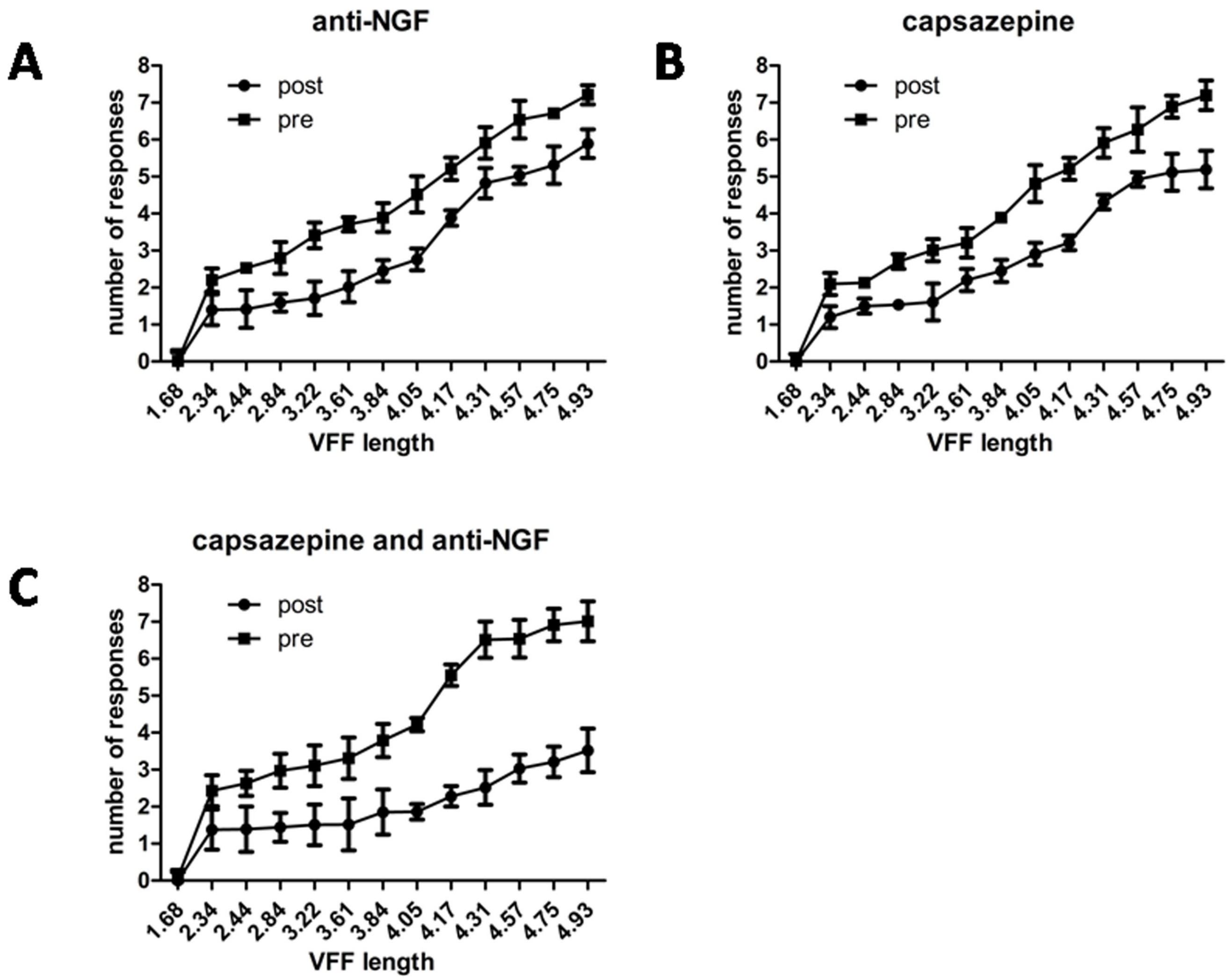

Supplementary Figure S6: Anti-EGF or/and capsazepine reduced the sensitivity to mechanical stimulation in mice. (**A**) Anti-NGF treatment attenuated behavioral responses. The neutralizing antibodies were intraperitoneally injected, and the sensitivity of the abdomen to mechanical stimulation was measured after 3 days. (**B**) Capsazepine treatment diminished behavioral responses. The methods are similar to those with anti-NGF. (**C**) Combining both anti-NGF and capsazepine obviously suppressed the pain-related behaviors.

Supplementary Table S1: Real-time PCR primer sequence

Gene	Primer Sequence
SHH	P1: 5′-GCTTCGACTGGGTGTACTACG-3′<br>P2: 5′-GCCACCGAGTTCTCTGCT-3′
BDNF	P1: 5′-TGGCTGACACTTTCGAACAC-3′<br>P2: 5′-CCTCATGGACATGTTTGCAG-3′
SP	P1: 5′-GACTCCTCTGACCGCTAC-3′<br>P2: 5′-AGACCTGCTGGATGAACT-3′
GDNF	P1: 5′-AGGTCACCAGATAAACAAGCGG-3′<br>P2: 5′-TCACAGGAGCCGCTGCAATATC-3′
TRPV1	P1: 5′-CCCATTGTGCAGATTGAGCAT-3′<br>P2: 5′-TTCCTGCAGAAGAGCAAGAAGC-3′
CGRP	P1:5′-AACCTTAGAAAGCAGCCCAGGCATG-3′<br>P2:5′-GTGGGCACAAAGTTGTCCTTCACCA-3′
NGF	P1: 5′-AAGGCTTTGCCAAGGACG-3′<br>P2: 5′-GTGATGTTGCGGGTCTGC-3′
GAPDH	P1: 5′-ACCACAGTCCATGCCATCAC-3′<br>P2: 5′-TCCACCACCCTGTTGCTGTA-3′
